# Compression of Bio-Signals Using Block-Based Haar Wavelet Transform and COVIDOA for IoMT Systems

**DOI:** 10.3390/bioengineering10040406

**Published:** 2023-03-24

**Authors:** Doaa Sami Khafaga, Eman Abdullah Aldakheel, Asmaa M. Khalid, Hanaa M. Hamza, Khaid M. Hosny

**Affiliations:** 1 Department of Computer Sciences, College of Computer and Information Sciences, Princess Nourah bint Abdulrahman University, P.O. Box 84428, Riyadh 11671, Saudi Arabia; dskhafga@pnu.edu.sa; 2 Information Technology Department, Faculty of Computers & Informatics, Zagazig University, Zagazig 44519, Egypt; asmaa.elhenawy@gmail.com (A.M.K.); hanaa_hamza2000@yahoo.com (H.M.H.); k_hosny@yahoo.com (K.M.H.)

**Keywords:** COVIDOA, compression, Haar wavelet, reconstruction, optimization, ECG

## Abstract

Background: Bio-signals are the essential data that smart healthcare systems require for diagnosing and treating common diseases. However, the amount of these signals that need to be processed and analyzed by healthcare systems is huge. Dealing with such a vast amount of data presents difficulties, such as the need for high storage and transmission capabilities. In addition, retaining the most useful clinical information in the input signal is essential while applying compression. Methods: This paper proposes an algorithm for the efficient compression of bio-signals for IoMT applications. This algorithm extracts the features of the input signal using block-based HWT and then selects the most important features for reconstruction using the novel COVIDOA. Results: We utilized two different public datasets for evaluation: MIT-BIH arrhythmia and EEG Motor Movement/Imagery, for ECG and EEG signals, respectively. The proposed algorithm’s average values for CR, PRD, NCC, and QS are 18.06, 0.2470, 0.9467, and 85.366 for ECG signals and 12.6668, 0.4014, 0.9187, and 32.4809 for EEG signals. Further, the proposed algorithm shows its efficiency over other existing techniques regarding processing time. Conclusions: Experiments show that the proposed method successfully achieved a high CR while maintaining an excellent level of signal reconstruction in addition to its reduced processing time compared with the existing techniques.

## 1. Introduction

**Figure 1:**
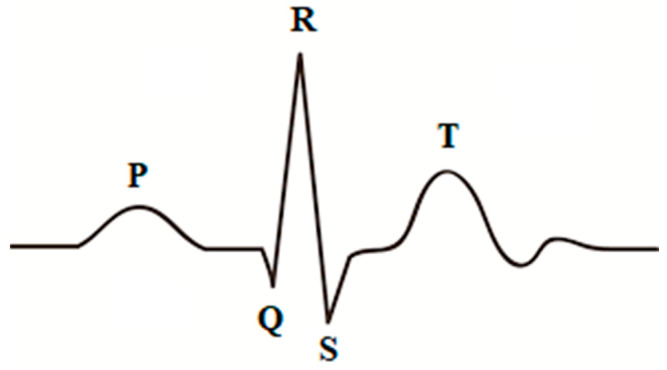
Single heartbeat.

**Figure 2:**
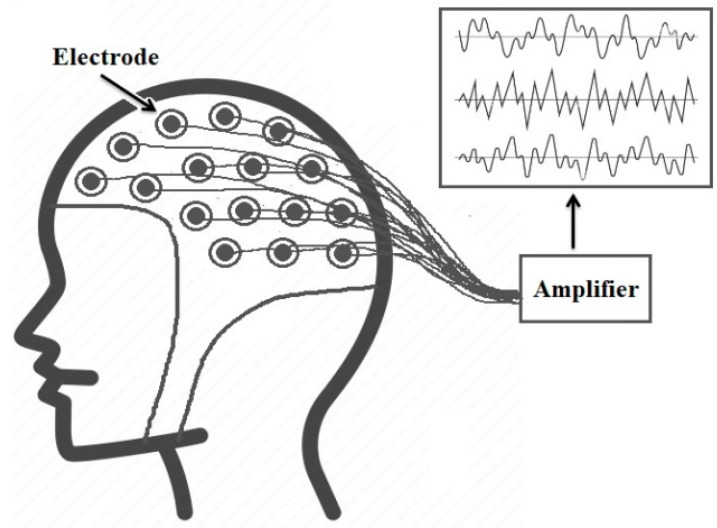
EEG recording [4].

Smart healthcare systems deal with massive amounts of medical data daily for healthcare monitoring and early detection and diagnosis of diseases [1]. Bio-signals are records of biological events inside the human body, such as a heartbeat or muscle contraction. These signals are used to detect whether there is a problem or disorder in a human organ. There are many kinds of bio-signals used for various clinical purposes, such as ECG, which is used for recording human heart activity, EEG for recording the electrical activity of the brain, EMG for evaluating the electrical activity of skeletal muscles, ERG for measuring the electrical responses of various cell types in the retina, and EGG for recording the myoelectrical signal generated by the movement of the smooth muscle of the stomach [2]. ECG and EEG are the most widely used bio-signals for diagnoses of cardiac and brain disturbances [3]. Three main elements represent a typical heartbeat signal: the P wave, which indicates depolarization of the atria, the QRS complex, which shows depolarization of the ventricles, and the T wave, which represents repolarization of the ventricles, as shown in Figure 1. On the other hand, EEG signals record the brain’s electrical activity. Several sensors are positioned on various parts of the scalp to record EEG signals, as shown in Figure 2. EEG signals help to identify various common diseases, such as epilepsy and autism spectrum disorder [4].

In smart healthcare systems, the bio-signals are recorded by sensors attached to the patient’s body and then digitized. The digital bio-signals are processed using digital computers or computer-based medical devices [5]. An effective compression method is a basic need in such systems to minimize the volume of medical data and enhance the transmission’s efficiency [6]. However, obtaining high compression ratios is insufficient for an efficient compression algorithm, and data quality must also be maintained since the loss of medical data could lead to misdiagnosis problems. For these reasons, we proposed an algorithm for efficient compression of bio-signals that can achieve very high CR (CR = 32) and preserve the diagnostic features of the input signal. This algorithm is based on a block-based HWT and COVIDOA. The HWT is used to obtain the features of the signal for these reasons [7]:

- The HWT can extract local spectral and temporal information simultaneously.
- Wavelet-based coding allows for progressive data transmission and is more robust to transmission and decoding failures.
- The HWT is conceptually simple and fast.
- The HWT is completely reversible and does not suffer from the edge effects that are an issue with other wavelet transformations.
- Block-based HWT and inverse transform can be performed by applying matrix multiplication.

We apply the HWT to the input signal and then select the best-fit coefficients to reconstruct the digital signal using COVIDOA according to a predefined objective function. The PRD is selected as the objective function so that the coefficients that lead to the minimum PRD values will be selected for reconstruction. The PRD is calculated as follows:

(1) $$\mathrm{PRD}\;\%=\sqrt{\frac{\sum_{x=0}^{N-1}{\left(f\left(x\right)-F\left(x\right)\right)}^{2}}{\sum_{x=0}^{N-1}f{\left(x\right)}^{2}}}\times 100$$

The F and f are reconstructed and original signals.

The rest of the paper is organized as follows: Concise literature is presented in Section 2. The explanation of calculating the HWT is discussed in Section 3. In Section 4, a brief overview of COVIDOA is presented. The proposed compression/decompression algorithm using HWT and COVIDOA is described in Section 5. Experiments, results, and discussion are presented in Section 6. The conclusion and the recommendation for future work are drawn in the last section.

## 2. Literature Review

Over the last few decades, various algorithms have been proposed to compress medical data. These algorithms are either lossless or lossy compression. The lossless compression methods can achieve small compression ratios with no data loss, while lossy algorithms achieve much higher compression ratios but some information will be lost [8]. Data quality is crucial in the medical field, and losing some features may significantly impact the diagnosis process. However, suppose the data loss is within an acceptable limit and does not affect the data’s visual appearance. In that case, lossy compression techniques will be a good choice due to the high compression ratio they can achieve [9]. In [10], an ASCII character-encoding-based lossless compression method was proposed. In [11], Chen and Wang used two Huffman coding tables to develop a useful lossless compression method to reduce the storage and transmission demands for ECG signals. This algorithm has the advantages of low cost and power consumption. Rzepka [12] used selective linear prediction to compress multi-channel ECG.

Most lossy compression algorithms are based on transform coding, where a specific transform is applied to the input signal, and some information is used to be discarded. In contrast, the others are used in the reconstruction process. The result of this process will not be identical to the original input, but it should be close enough according to the application’s purpose. The most popular transform-based compression techniques involve the DCT [13], DWT [7], and moment-based transform [14]. For bio-signal compression, Batista et al. [15] utilized Golomb–Rice coding with optimum DCT coefficients to compress ECG signals. They used the well-known MIT-BIH Arrhythmia database to evaluate their algorithm, where CR of 10.4:1 and PRD ≅ 2.5% were achieved. Jha and Kolekar [16] proposed another DCT-based algorithm to compress ECG signals. They employed DOST and dead-zone quantization to transform coefficients. Recent work includes the technique proposed in [17] for assessing compressed and decompressed ECG databases. The proposed algorithm used DCT, 16-bit quantization, run-length encoding for compression, and convolution neural network for classification. The obtained CR was 2.56, and the classification accuracies were 0.966 and 0.990 for the compressed and decompressed databases, respectively. Further, Pal et al. [18] proposed a compression algorithm for 2D ECG signals based on the combination of DCT and embedded zero-tree wavelet. The results showed that the suggested approach could raise the sparsity of the transform domain, which boosts compression effectiveness with a small degradation in reconstruction quality. Other DCT-based signal compression algorithms are proposed in [19,20].

The WT is a powerful tool for signal analysis because of its compact representation of signals and images, and its most popular applications are denoising and compression of signals [21]. Recently, OMs, such as Tchebichef and Hahn moments, have been used in signal reconstruction and compression due to their ability to represent signals [22]. Signal compression techniques based on orthogonal moments are presented in [23,24]. It is observed from the state of the art that the wavelet-based algorithms provide superior performance compared to the other compression methods [21]. Jha and Kolekar [25] used the DWT to select an appropriate mother wavelet to compress the ECG signal while guaranteeing quality. The same authors employed EMD and DWT to develop another ECG compression algorithm [26]. Based on the obtained results, it is noticed that the suggested method performs better than several current ECG compressors. Singhai et al. [27] used DWT and PSO to design a compression algorithm where the PSO selects threshold values and the optimal wavelet parameters. The compression ratio obtained using this algorithm was 28.43 at PRD = 2.63. Kolekar et al. [28] proposed an ECG compression technique based on the modified run-length encoding of wavelet coefficients. The proposed approach used dead-zone quantization for WT coefficients, and the obtained coefficients were encoded using modified run-length encoding.

Shi et al. [29] proposed a new ECG compression method based on a binary convolutional auto-encoder (BCAE) equipped with residual error compensation (REC). The proposed method aimed to achieve efficient ECG compression through deep learning while ensuring high signal quality. The performance is tested using several measures, such as PRD, QS, SNR, and CR. The average performance in CR, PRD, NPRD, and SNR is 17.18, 3.92, 6.36, and 28.27 dB, respectively, for 48 ECG records. The achieved results in CR and PRD are 117.33 and 7.76, respectively. Recently, Singhai et al. [30] presented an algorithm for ECG compression based on DWT and nature-inspired optimization algorithms. The algorithm used the optimization algorithm to find the optimal wavelet design parameter values and optimal threshold levels. The results show the capability of this technique to provide high compression ratios with high signal quality.

Lossy compression depends on using only some features and omitting others in return for reduced size. The question is, therefore, which features should be selected and which should not? The answer should be that the features that contain the most important clinical features and lead to the highest reconstruction quality should be selected, and the remaining features should be neglected. An optimization algorithm would be very helpful in selecting the most important feature subset. Motivated by the simplicity and efficiency of the HWT in signal and image processing and the efficiency of COVIDOA in solving various optimization tasks, we utilized the HWT in combination with COVIDOA to develop an efficient compression algorithm for bio-signals. In this approach, the signal is transformed using the HWT and then the best feature subset from the wavelet coefficients that should be used for reconstructing the signal will be selected with the help of COVIDOA.

## 3. HWT

This section explains applying a fast block-based HWT to a one-dimensional signal. Suppose the signal is divided into K blocks denoted by Bi, i = 1, 2, 3, …, K, where Bi is the ith block of size 1 × N. The following formula can perform the forward HWT for each block:

(2) $$T={\mathrm{BA}}^{T}$$

where B is the signal block and A is the Haar matrix.

The Haar matrix can be obtained using the following formula [31]:

(3) $$A_{0}\left(x\right)=\frac{1}{\sqrt{N}},\;\left(0\leq x\leq X\right)$$

(4) $$A_{1}\left(x\right)=\frac{1}{\sqrt{N}}\left\{\begin{array}{c} 1,\;\;\;\;\;0\leq x\leq X,\\ -1,\;\;\;\;\frac{X}{2}\leq x\leq X,\\ 0,\;\;\;\;\;\;\mathrm{otherwise}, \end{array}\right.$$

and

(5) $$A_{i}\left(x\right)=\frac{1}{\sqrt{N}}\left\{\begin{array}{c} 2^{\frac{j}{2}},\;\;\;\;\frac{k-1}{2^{j}}X\leq x\leq \frac{k-\frac{1}{2}}{2^{j}}X,\\ -2^{\frac{j}{2}},\;\;\;\;\;\;\frac{k-\frac{1}{2}}{2^{j}}X\leq x\leq \;\frac{k}{2^{j}}X,\\ 0,\;\;\;\;\;\;\;\;\;\;\;\;\;\;\;\;\;\;\;\;\;\;\;\;\;\;\mathrm{otherwise}, \end{array}\right.$$

where i = 1, 2, …, N − 1, $N=2^{j}\left(j=0,\;1,\;\ldots ,\;J\right)\;\mathrm{refers}\;\mathrm{to}\;\mathrm{the}\;\mathrm{wavelet}\;\mathrm{level}.$ J represents the resolution. j and k represent the integer decomposition of the index i, where i = N + k − 1 and k = 1, 2, …,$\;2^{j}$. $A_{0}\left(x\right)\;\mathrm{represented}\;\mathrm{the}\;\mathrm{scaling}\;\mathrm{function}$ while $A_{1}\left(x\right)$ is the mother wavelet function. The other remaining Haar wavelet functions can be obtained from the mother function $A_{1}\left(x\right)$ by applying translation and dilation processes.

According to the previous formula, the kernel matrix for the HWT can be generated as follows:

(6) $$A=\left[\begin{array}{cccc} A_{0}\left(1\right) & A_{0}\left(2\right) & \ldots & A_{0}\left(N-1\right)\\ A_{1}\left(1\right) & A_{1}\left(2\right) & \ldots & A_{1}\left(N-1\right)\\ A_{2}\left(1\right) & A_{2}\left(2\right) & \ldots & A_{2}\left(N-1\right)\\ \vdots & \vdots & \ddots & \vdots \\ A_{N-1}\left(1\right) & A_{N-1}\left(2\right) & \ldots & A_{N-1}\left(N-1\right) \end{array}\right]$$

According to N, the Haar wavelet kernel matrix can be of size 4 × 4, 8 × 8, 16 × 16, 32 × 32, or 64 × 64. The 8 × 8 Haar wavelet matrix is as follows:

(7) $$AA_{8\times 8}=\left[\begin{array}{cccccccc} \frac{1}{\sqrt{8}} & \frac{1}{\sqrt{8}} & \frac{1}{\sqrt{8}} & \frac{1}{\sqrt{8}} & \frac{1}{\sqrt{8}} & \frac{1}{\sqrt{8}} & \frac{1}{\sqrt{8}} & \frac{1}{\sqrt{8}}\\ \frac{1}{\sqrt{8}} & \frac{1}{\sqrt{8}} & \frac{1}{\sqrt{8}} & \frac{1}{\sqrt{8}} & \frac{-1}{\sqrt{8}} & \frac{-1}{\sqrt{8}} & \frac{-1}{\sqrt{8}} & \frac{-1}{\sqrt{8}}\\ \frac{1}{\sqrt{4}} & \frac{1}{\sqrt{4}} & \frac{-1}{\sqrt{4}} & \frac{-1}{\sqrt{4}} & 0 & 0 & 0 & 0\\ 0 & 0 & 0 & 0 & \frac{1}{\sqrt{4}} & \frac{1}{\sqrt{4}} & \frac{-1}{\sqrt{4}} & \frac{-1}{\sqrt{4}}\\ \frac{1}{\sqrt{2}} & \frac{-1}{\sqrt{2}} & 0 & 0 & 0 & 0 & 0 & 0\\ 0 & 0 & \frac{1}{\sqrt{2}} & \frac{-1}{\sqrt{2}} & 0 & 0 & 0 & 0\\ 0 & 0 & 0 & 0 & \frac{1}{\sqrt{2}} & \frac{-1}{\sqrt{2}} & 0 & 0\\ 0 & 0 & 0 & 0 & 0 & 0 & \frac{1}{\sqrt{2}} & \frac{-1}{\sqrt{2}} \end{array}\right]$$

The original signal block B can then be reconstructed from its transform by applying the inverse Haar transform as follows:

(8) AR=TA

T and R are the reconstructed signal block and the transform coefficients.

## 4. COVIDOA

COVIDOA is a recent metaheuristic inspired by the replication life cycle of the novel Coronavirus particles inside the human body [32]. COVIDOA is divided into four stages as follows:

- Virus entry and uncoating

The virus particle tries to enter the human body cell through a special structural protein called spike protein. After entry, the virus genome is uncoated inside the cell.

b. Virus replication

The virus uses the frameshifting technique to generate millions of copies to hijack as many human cells as possible. The most popular frameshifting technique is +1 frameshifting, in which the elements of the parent sequence are moved forward by one step, resulting in losing the first element in the parent sequence, which will be replaced by a random value between lb and ub as follows:

(9) $$V_{t}\left(1\right)=\mathrm{rand}\;\left(\mathrm{lb},\mathrm{ub}\right),$$

(10) $$V_{t}\left(2:D\right)=P\left(1:D-1\right)$$

The P is the parent sequence; the $V_{t}$ is the generated viral protein number t; lb and ub are the lower and upper bounds; D is the problem dimension.

c. Virus mutation

The virus tries to mutate to hijack the immune system as follows:

(11) $$Z_{i}=\left\{\begin{array}{c} r\;\;\;\;\;\mathrm{if}\;\mathrm{rand}\left(0,1\right)<MR\\ X_{i}\;\;\;\;\;\;\;\;\;\;\;\;\;\;\;\;\;\;\;\;\;\mathrm{otherwise} \end{array}\right.$$

Z and X are the mutated and non-mutated solutions, and i = 1, …, D. r is a random number in a range [lb, ub]. MR is the mutation rate, which has a value from 0.005 to 0.5.

d. New virion formation and release

The previous steps generate many new virus particles called virions, which are then released from the infected cell and directed to new cells. The pseudocode of COVIDOA is shown in Algorithm 1.

**Algorithm 1** Pseudocode of COVIDOA.Set initial values of the following parameters: Dimension (D), population size (popSize), maximum number of iterations (MaxItr), number of proteins, shifting number, and mutation rate (MR).For (i = 1: I ≤ nPop) do   Generate initial random population.   Evaluate the fitness function for all solutions in the population.End forOrder solutions ascendingly according to fitness function.Set the first solution as the optimum solution.Set t = 1Repeat   For (i = 1: I ≤ nPop) do     Select a parent solution P,     For (k = 1: I ≤ number of proteins) do        Generate protein V_k_ from parent solution P using Equations (9) and (10).     End for     Apply uniform crossover between the generated proteins to generate new virion (new solution).     if (rand (0,1) < MR) then        Mutate the new solution using Equation (11).     End if   End forUntil t ≥ MaxItr

## 5. The Proposed Compression/Decompression Algorithm

In the proposed compression algorithm, we utilized a simplified block-based HWT to obtain the Haar coefficients for the signal; then, the COVIDOA is used to select a subset of the coefficients needed for signal reconstruction. The size of the selected subset is determined according to the desired compression ratio (CR) using the following formula:

(12) $$\mathrm{SS}\;=\;\mathrm{Round}\;\left(1-\frac{\mathrm{CR}}{100}\right)\times \;N$$

where SS refers to the size of a selected subset of coefficients, CR is the desired compression ratio, and N is the signal block size. The following steps can summarize the proposed compression algorithm:

1. The signal is split up into blocks of size 1 × N; N can be 8, 16, 32, or 64.
2. The required subset of the size of the coefficients is calculated using Equation (12).
3. The Haar wavelet kernel matrix is calculated using Equations (3)–(5).
4. The parameters of COVIDOA are set as follows: D = SS; population size (popSize) = 30; the maximum number of iterations (MaxItr) = 50; the number of proteins = 2; shifting number = 1; mutation rate (MR) = 0.5.
5. For each signal block
  - Calculate the block-based HWT to obtain the Haar coefficients using Equation (2).
  - COVIDOA is used to select the optimal coefficients according to the PRD objective function using Equation (1) as follows:
    - Generate an initial random population of solutions and compute the objective function for each solution.
    - Select parent solution using tournament selection and apply the frameshifting technique to generate several proteins using Equations (9) and (10).
    - Apply crossover between the generated proteins to generate a new virion.
    - Apply mutation to the previously generated solution to obtain a new mutated solution.
    - Replace the new solution with the parent solution if the new solution is fitter than the parent. Otherwise, the parent solution remains.
    - Repeat steps ii to v until the MaxItr is reached.
    - Select the optimal solution achieved so far.
  - From the coefficient obtained in step a, only the coefficients whose positions correspond to the values in the optimum solution are selected, and the remaining coefficients are ignored (set to zero).
  - Apply the inverse transform to the optimum coefficients obtained in the previous step to obtain the reconstructed signal block using Equation (8).
6. Concatenate the reconstructed blocks to obtain the reconstructed signal.
7. Evaluate the algorithm’s performance using CR, PRD, SSIM, and QS metrics.

**Figure 3:**
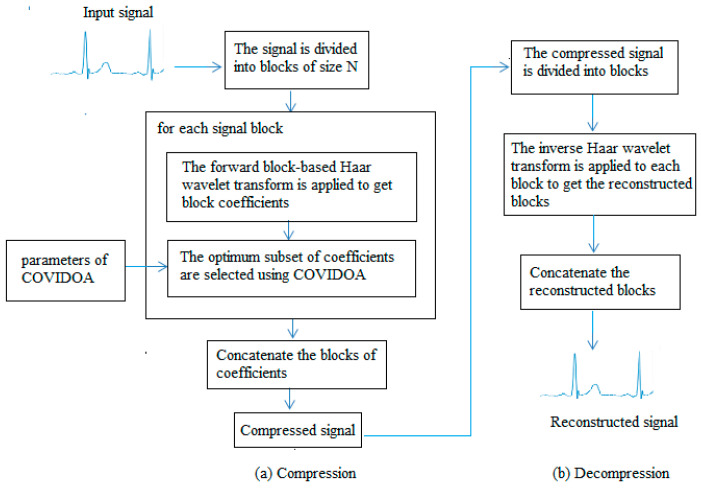
Compression and Decompression processes of the suggested algorithm.

A diagram of the proposed compression/decompression approach is displayed in Figure 3.

## 6. Results

This section provides a brief overview of the utilized datasets, the evaluation criteria, and the numerical results and discussions about the experiments performed by the proposed algorithm as follows:

### 6.1. Datasets

Two separate bio-signal datasets are utilized to evaluate the performance of the proposed compression algorithm. The MIT-BIH arrhythmia dataset is used for testing ECG compression [33]. Further, 25 ECG records that contain cardiac information for a group of volunteers are selected for evaluation. The selected signals have a sampling rate of 360 samples per second and a resolution of 11 bits. The second dataset is the Motor Movement/Imagery Dataset, used for testing EEG compression [34]. This dataset contains over 1500 one- and two-minute EEG recordings collected from 109 volunteers. Twenty single-channel EEG signals are selected for testing. The sampling frequency of the selected EEG signals is 160 samples per second.

### 6.2. Evaluation Criteria

The compression ratio and the reconstructed image quality must be measured to evaluate the proposed algorithm’s performance. Various metrics are utilized for evaluation as follows:

- CR

The achieved CR can be measured as follows:

(13) $$\mathrm{CR}=\frac{N_{O}}{N_{R}}$$

N_O_ and N_R_ represent the number of bits in the original and reconstructed signals.

- PRD %

This metric is used to quantify the difference between the original and reconstructed signals as follows:

(14) $$\mathrm{RD}=\sqrt{\frac{\sum {\left(f\left(x\right)-F\left(x\right)\right)}^{2}}{\sum f{\left(x\right)}^{2}}}\times 100$$

- NCC

NCC is used to measure the correlation between two signals. Its value ranges from 1 to −1, where 1 represents a complete positive correlation and −1 represents a complete negative correlation. The NCC between the original signal f(x) and reconstructed signal F(x) is calculated as follows:

(15) $$\mathrm{NCC}=\frac{\sum (f\left(x\right)-\bar{f}\left(x\right))(F\left(x\right)-\bar{F}\left(x\right))}{\sqrt{\sum {\left(f\left(x\right)-\bar{f}\left(x\right)\right)}^{2}(F\left(x\right)-\bar{F}{\left(x\right)}^{2})}}$$

$\bar{f}\left(x\right)$ and $\bar{F}\left(x\right)$ are the mean values for the original and reconstructed signals, respectively.

- QS

QS is used to measure the performance of the compression algorithm. The higher the value of QS, the better the algorithm’s performance. QS can be calculated as follows:

(16) $$\mathrm{QS}=\frac{\mathrm{CR}}{\mathrm{PRD}}$$

For a fair comparison, the proposed and competing algorithms were tested on a PC with the following specifications: Intel(R) Core(TM) i7-1065G7 CPU, 8 GB RAM, Windows 10 operating system, and MATLAB R2016a development environment.

### 6.3. Numerical Results and Discussion

**Figure 4:**
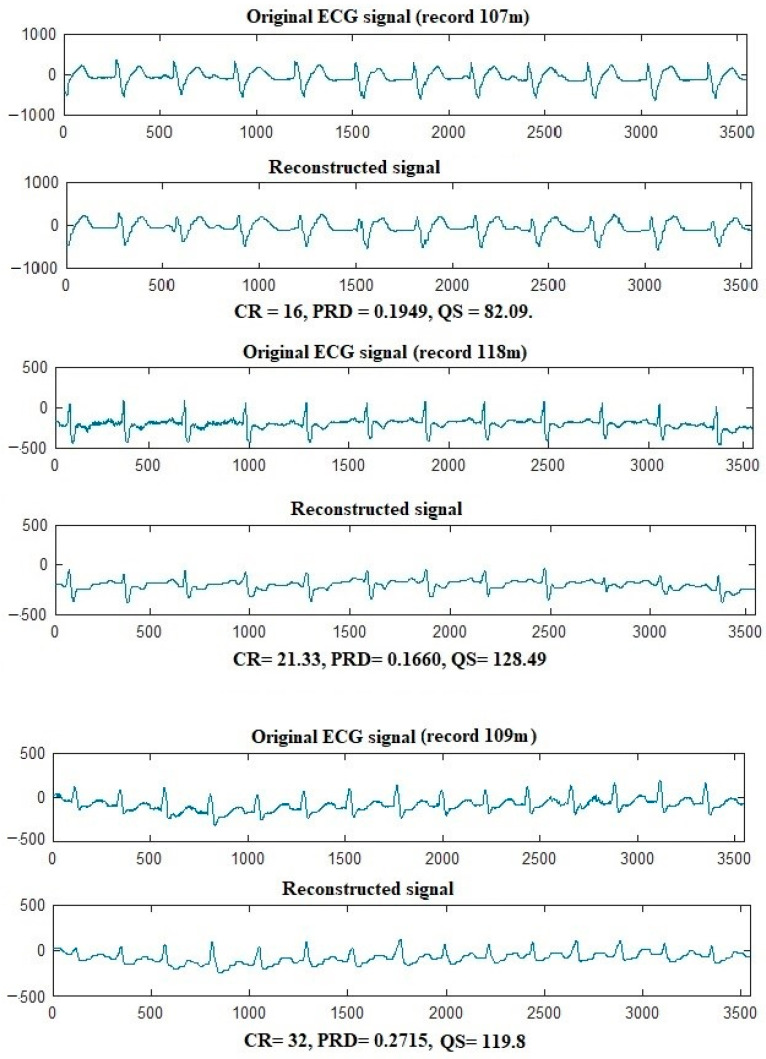
Here, 107, 118, and 109 ECG records compression with the suggested algorithm.

This section presents the numerical outcomes of the suggested ECG and EEG compression algorithm. For ECG compression, 25 records from the MIT-BIH arrhythmia dataset are compressed using the proposed approach, and the results are shown in Table 1. It can be noticed from the table that the proposed approach has efficient compression performance as it can achieve high CR (CR = 32) with excellent signal reconstruction quality (PRD = 0.2665 and NCC = 0.9436). Additionally, the high-quality score results prove the efficient overall performance of the proposed compression approach. For example, records 108, 112, 117, 118, and 121 have the highest QS results 119, 166, 110, 142, and 211. The average results of the proposed approach in terms of CR, PRD, NCC, and QS are 18.06, 0.2470, 0.9467, and 85.366. Figure 4 shows examples of ECG signals after compression and decompression by the proposed algorithm. The excellent quality of the reconstructed signals is clear from the figure, as the reconstructed signals are very similar to the original.

**Figure 5:**
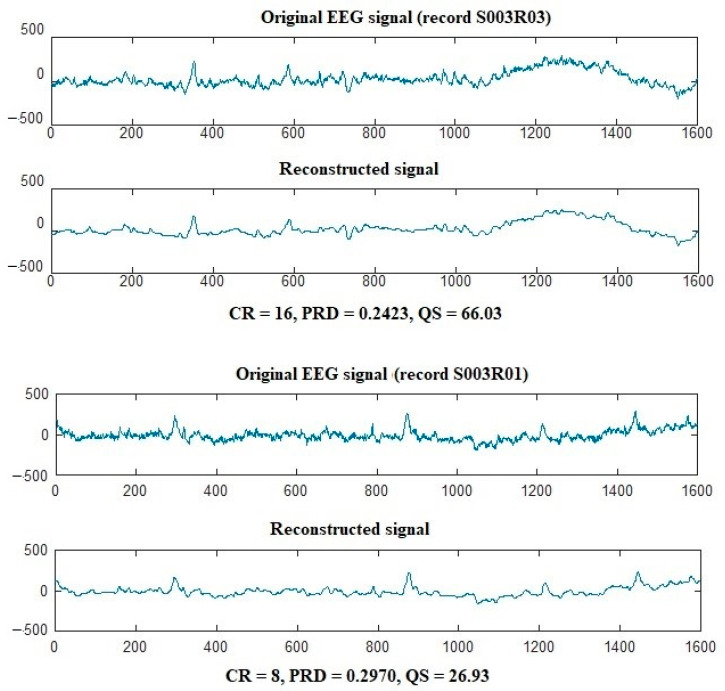
Compression of S003R03 and S003R01 EEG records using the proposed algorithm.

For EEG compression, 20 single-channel EEG signals from the Motor Movement/Imagery Dataset are compressed and decompressed by the proposed approach. The results in terms of CR, PRD, NCC, and QS and displayed in Table 2. It is shown from the table that the proposed algorithm can achieve high CR values while maintaining signal quality for EEG signals. The best EEG compression results for the proposed algorithm are 16, 0.2423, 0.9725, and 66.033 for CR, PRD, NCC, and QS, respectively. The proposed algorithm can achieve average CR, PRD, NCC, and QS values of 12.6668, 0.4014, 0.9187, and 32.4809, respectively. Figure 5 shows samples of EEG signals before and after compression by the proposed algorithm.

A comparison with some existing approaches [22,23,24] to prove the efficiency of the proposed compression algorithm over the state-of-the-art techniques is conducted in terms of CR, PRD, and QS, as shown in Table 3. It is shown from the table that with the same CR, the proposed approach achieves the minimum PRD and maximum QS values, which demonstrates the proposed algorithm’s superiority to the competing methods.

In remote healthcare monitoring systems, the compression speed is very important as a higher compression speed means reduced energy consumption. Along with the evaluation measures already mentioned, we also compared the other techniques in processing time. Table 4 and Table 5 show the processing time in seconds for the proposed and existing compression techniques for ECG and EEG signals. The tables demonstrate that the proposed algorithm has a lower processing time than the other techniques in most cases. However, in some cases, the technique in [24], which uses Tchebichef moments with the ABC algorithm, has the lowest processing time, especially in higher compression ratios. We conclude from previous experiments that the proposed compression algorithm achieves excellent performance for bio-signal compression.

## 7. Conclusions

In this paper, an efficient compression algorithm is proposed for bio-signals. Because of the simplicity and efficiency of the HWT in extracting signal information, the proposed technique used the block-based HWT to extract the signal’s features. The novel COVIDOA selects the best wavelet coefficient subset to achieve the desired compression ratio. The optimum coefficient subset is selected according to a selected objective function, PRD. The subset of coefficients that achieves the minimum PRD value is selected and is considered the optimum and selected for signal reconstruction. The MIT-BIH arrhythmia and Motor Movement/Imagery datasets are used to test the performance of the proposed algorithm in ECG and EEG signal compression. The results showed that the proposed compression approach could achieve high CRs while maintaining signal quality. Comparing existing compression algorithms is conducted according to CR, PRD, NCC, QS, and processing time. The comparison proved the superiority of the proposed algorithm in ECG and EEG compression.

Future work may include applying the proposed algorithm for other bio-signals, such as EMG, ERG, and EGG. Further, the proposed approach may be applied to 2D and 3D medical image compression to minimize the storage and transmission capabilities required by healthcare systems.

## Figures and Tables

**Table 1:** Result of ECG signal compression using the proposed algorithm.

Signal	Metric			
	CR	PRD	NCC	QS
100	25.6000	0.2764	0.9305	92.6194
101	21.3	0.2951	0.9173	72.2034
102	21.3	0.3700	0.9399	57.5676
103	10.6667	0.2628	0.9734	40.7137
104	16	0.2730	0.9833	58.6081
105	16	0.3051	0.9763	52.4590
106	8	0.2336	0.9890	34.2466
107	10.6667	0.1832	0.9909	58.2242
108	21.333	0.1790	0.9689	119.1788
109	32	0.2665	0.9436	119.8951
111	10.6667	0.2471	0.9805	43.1850
112	16	0.0968	0.9250	166.6667
113	10.6667	0.2584	0.9739	41.3438
114	21.3333	0.5272	0.9238	40.4647
115	21.333	0.3017	0.8993	70.8738
116	18.2857	0.2845	0.9188	64.3863
117	16	0.1453	0.9158	110.1170
118	21.333	0.1501	0.9455	142.2222
119	16	0.1511	0.9480	105.8901
121	21.3	0.1008	0.9452	211.3095
122	16	0.1660	0.9653	96.3855
123	21.3	0.1765	0.8928	120.6799
124	21.3	0.1856	0.9214	114.7629
200	21.3	0.3895	0.9301	54.6855
202	16	0.3518	0.9689	45.4804
Average	18.06	0.2470	0.9467	85.366

**Table 2:** Result of EEG signal compression using the proposed algorithm.

Signal	Metric			
	CR	PRD	NCC	QS
S001R01	8	0.4259	0.9145	18.7838
S001R02	10.6667	0.4440	0.8993	24.0241
S001R03	10.6667	0.4271	0.9257	24.9747
S001R04	16	0.3911	0.9207	40.9103
S001R05	10.6667	0.4481	0.8986	23.8027
S001R06	16	0.4344	0.9085	36.8324
S001R07	8	0.3580	0.9393	22.3464
S001R08	10.6667	0.4130	0.9224	25.8274
S001R09	10.6667	0.4134	0.9187	25.8024
S001R10	16	0.4116	0.9161	38.8727
S001R11	16	0.3595	0.9429	44.5063
S001R12	10.6667	0.4148	0.9159	25.7153
S001R13	8	0.3540	0.9381	22.5989
S001R14	16	0.3424	0.9427	46.7290
S002R01	16	0.4884	0.8829	32.7600
S002R02	10.6667	0.4436	0.8907	24.0458
S002R05	16	0.4753	0.8522	33.6629
S003R01	16	0.4329	0.9105	36.9600
S003R03	16	0.2423	0.9725	66.033
S003R05	10.6667	0.3098	0.9623	34.4309
Average	12.6668	0.4014	0.9187	32.4809

**Table 3:** Comparison with other techniques.

	Algorithm	Metric			
		CR	PRD	NCC	QS
100 m	Proposed	**25.6000**	**0.2821**	**0.9005**	**90.7801**
	Ref. [24]	16	0.4058	0.5068	39.4283
	Ref. [22]	10.6667	0.6993	0.4181	15.2534
101	Proposed	**21.3**	**0.2951**	**0.9173**	**72.2034**
	Ref. [24]	21.3	0.4411	0.5791	48.2884
	Ref. [22]	21.3	0.9085	0.3474	23.4452
112	Proposed	**16**	**0.0968**	**0.9190**	**166.6667**
	Ref. [24]	16	0.1930	0.6022	82.9016
	Ref. [22]	12.8000	0.9045	0.1599	14.1515
117	**Proposed**	**16**	**0.1453**	**0.8954**	**110.1170**
	Ref. [24]	16	0.1903	0.6946	84.0778
	Ref. [22]	16	0.9120	0.1919	17.5439
121	**proposed**	**21.3**	**0.1008**	**0.9352**	**211.3095**
	Ref. [24]	21.3	0.2403	0.5872	88.6392
	Ref. [22]	21.3	0.9291	0.1553	22.9254
S001R14	**Proposed**	**16**	**0.3424**	**0.9427**	**46.7290**
	Ref. [24]	16	0.3817	0.9317	41.9177
	Ref. [22]	16	0.6630	0.7470	24.1327
S003R03	**Proposed**	**16**	**0.2423**	**0.9725**	**66.033**
	Ref. [24]	16	0.3368	0.9686	47.5059
	Ref. [22]	16	0.6574	0.7365	24.3383

**Table 4:** Processing time of the proposed and existing techniques in seconds for ECG compression.

Signal	CR	8			10			16		
	Algorithm	Proposed	Ref. [24]	Ref. [22]	Proposed	Ref. [24]	Ref. [22]	Proposed	Ref. [24]	Ref. [22]
100	Time(s)	**16.8569**	20.50271	43.0804	5.9904	**5.2764**	12.7438	5.1200	**4.9127**	10.5913
101	Time(s)	**9.3482**	18.44711	24.0403	**5.3523**	5.31273	10.3791	**5.22571**	5.9275	9.9437
102	Time(s)	**20.92532**	22.2531	42.5343	**14.40836**	14.8672	21.5439	**12.74184**	15.4386	18.3288

**Table 5:** Processing time of the proposed and existing techniques in seconds for EEG compression.

Signal	CR	8			10			16		
	Algorithm	Proposed	Ref. [24]	Ref. [22]	Proposed	Ref. [24]	Ref. [22]	Proposed	Ref. [24]	Ref. [22]
**S003R03**	Time(s)	**19.84490**	22.29458	60.8584	**10.72566**	17.24832	18.9420	**6.588784**	8.447046	8.36956
**S001R06**	Time(s)	**19.05750**	32.05123	80.70620	**11.41363**	27.05330	34.86599	**10.18366**	17.933577	36.61682
**S002R01**	Time(s)	**22.52732**	45.5811	91.5449	**16.21887**	25.7678	44.4210	**15.2215**	15.93446	32.3308

## Data Availability

The data is publicly available at: https://archive.physionet.org/physiobank/.
